# Supplementary material for: A GABAergic Maf-expressing interneuron subset regulates the speed of locomotion in Drosophila
Source: Nat Commun. 2019 Oct 22;10:4796. doi: 10.1038/s41467-019-12693-6 (PMC6805931; doi:10.1038/s41467-019-12693-6)
Supplement: Supplementary file 1 — Supplementary Information [file 41467_2019_12693_MOESM1_ESM.pdf]

A GABAergic Maf-expressing interneuron subset regulates the speed of locomotion in *Drosophila*

Babski et al.

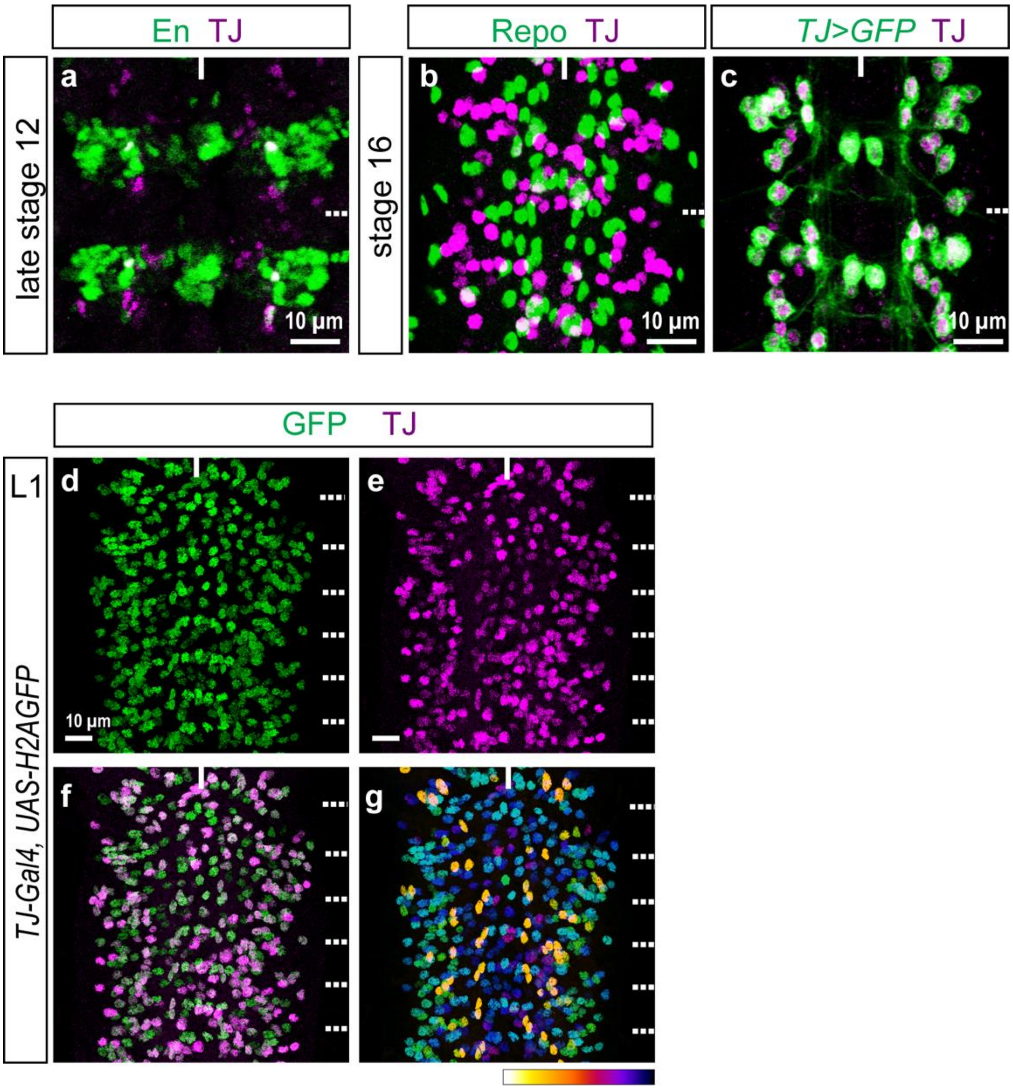

Supplementary Figure 1 TJ is specifically expressed in post-mitotic neurons in *Drosophila* embryo CNS. **a** Staining of stage 12 embryo VNC for TJ (magenta) and Engrailed (En) (green). TJ starts to be expressed at stage 12. **b** Staining of stage 16 embryo VNC for TJ (magenta) and glial marker Repo (green). TJ expression is restricted to neurons and excluded from glial cells. This projection view of several dorso-ventral confocal sections gives rise to some overlay between TJ^{+} and Repo^{+} nuclei (white) but no perfect overlap between these 2 types of nuclei can be found. **c** Staining of stage 16 embryo VNC for TJ (magenta) and GFP driven by *TJ-Gal4* (green). **d-f** Staining of a L1 VNC for TJ (magenta) and GFP driven by *TJ-Gal4* (green). In all embryonic and larval stages examined *TJ-Gal4* faithfully reports the expression of TJ (as seen with the antibody). Note that the levels of TJ protein vary from neurons to neurons. **g** 3D in depth color projection showing the respective dorsal to ventral locations of TJ+ neurons. Color code reads as follow: Yellow indicate dorsal most positions while dark blue ventral most locations. **In all panels**: Dashed lines on the right-hand side of the panels indicate segment boundaries and the full line the midline. **a-c** Two segments are shown; **g** six full segments can be visualized. Anterior part of the VNC is up.

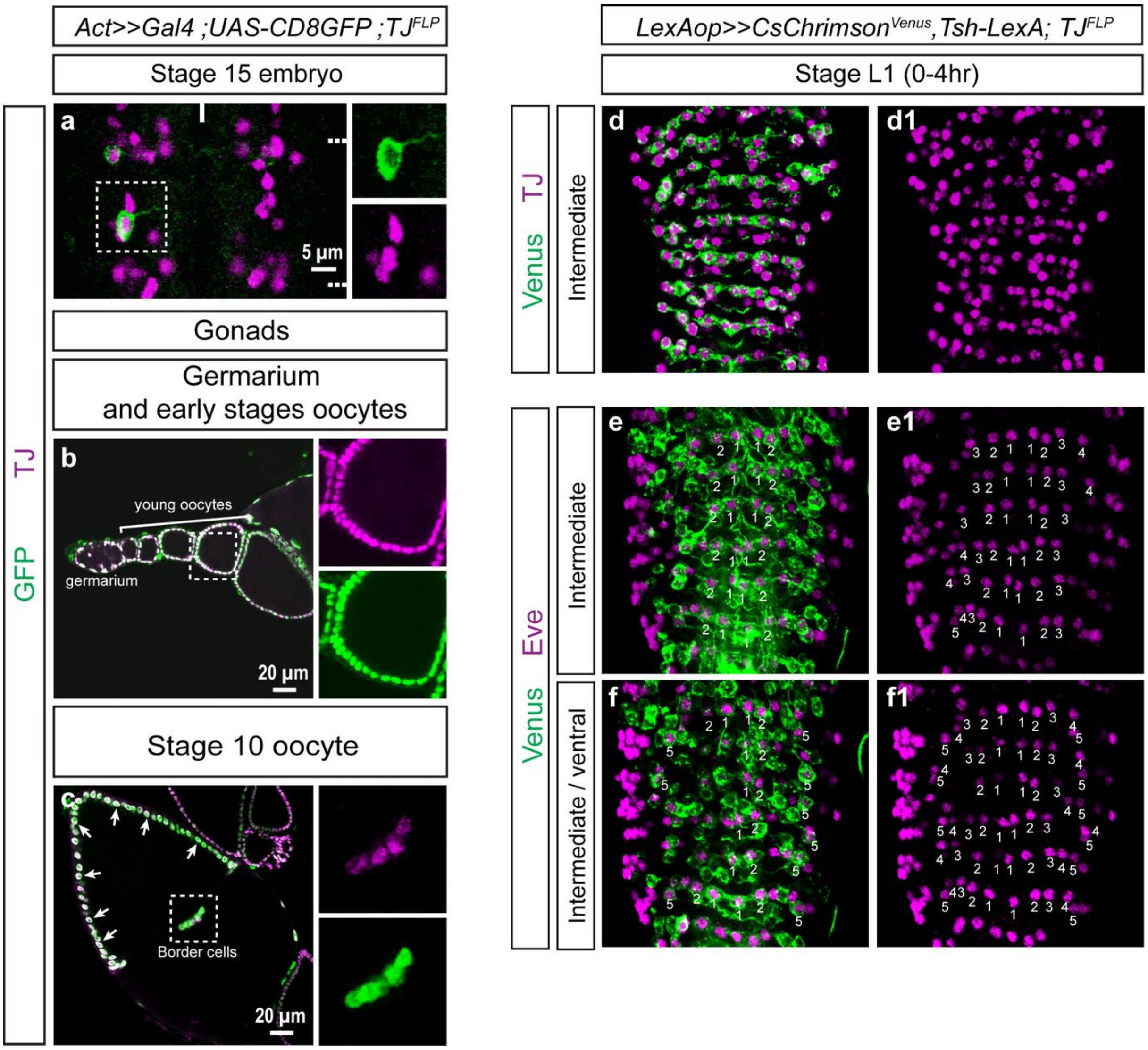

Supplementary Figure 2 Monitoring *TJ-FLP* activity using 2 distinct genetic approaches. **a-c** Staining for TJ (magenta) and GFP (*TJ-FLP* expressing cells; green) using *TJ-FLP* in combination with *Act>>Gal4, UAS-CD8GFP* in stage 15 embryonic VNC **a** and in adult female gonads **b, c**. The first recombined cells expressing GFP in the VNC can be detected by embryonic stage 15. In female gonads: germarium and early stages oocytes (**b**) and stage 10 oocyte (**c**) are shown. Cell types well characterised for their expression of TJ (follicular cells, arrows; border cells, circled) express the flippase. **d** Using *TJ-FLP* in combination *with LexAop>>CsChrimson-Venus, Tsh-LexA* revealed that 74,5% of the TJ-expressing neurons (n=852 TJ$^+$ neurons counted) have already recombined in young L1 larvae (0-4hr old). **e** Staining for Eve (magenta) allowing for the identification of U/CQ MNs revealed that Eve$^+$/TJ$^+$ MNs (namely U1, U2 and U5) are in 98% of the cases efficiently recombined in 0-4hr old larvae (n=48 hemisegments analysed). Close examination of these preparations also revealed that U3 and U4 (both TJ$^-$) have not been recombined, thus highlighting the high specificity of *TJ-FLP*. Anterior part of the VNC is up.

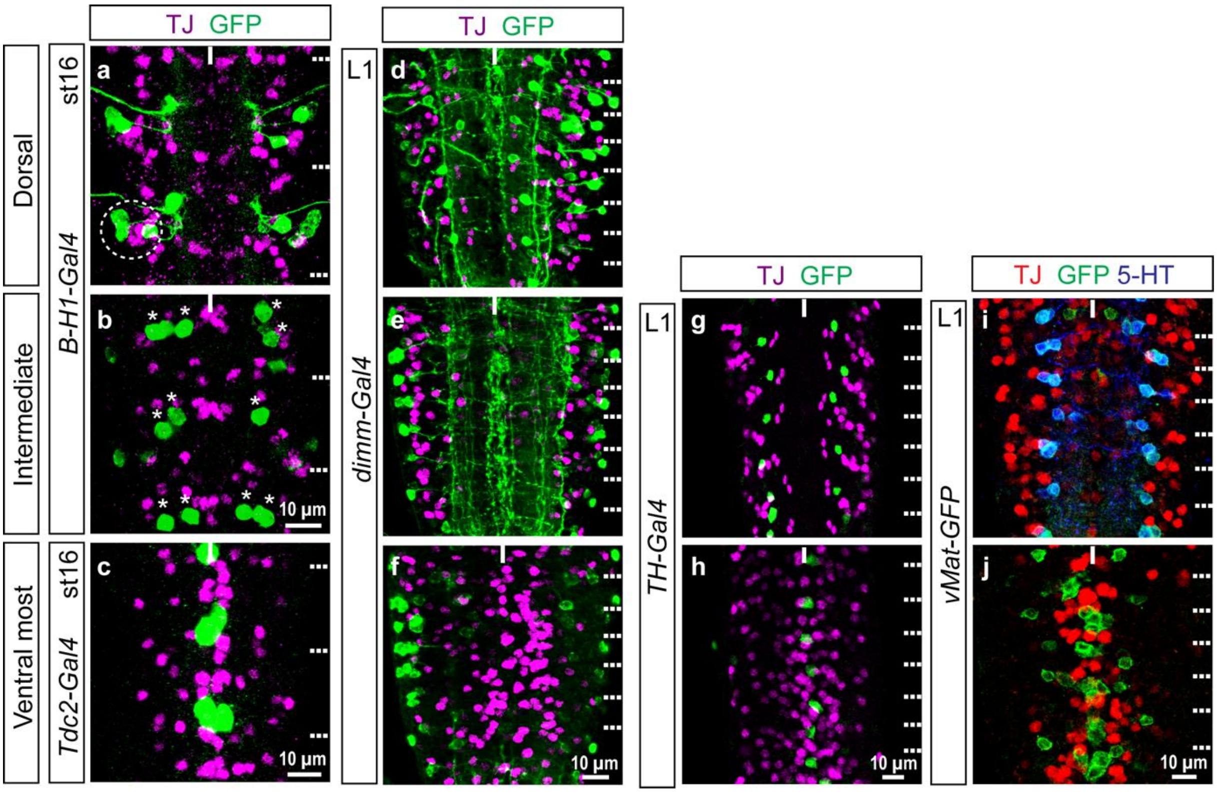

Supplementary Figure 3 TJ is excluded from monoaminergic and peptidergic neuronal populations. **a, b** Representative views of two segments of stage 16 embryonic VNC stained for TJ (magenta) and GFP labelling SNa MNs (green, *UAS-myrGFP* driven by *B-H1-gal4*). TJ is excluded from SNa MNs (**a**, circle). **c** Representative views of two segments of stage 16 embryonic VNC stained for TJ (magenta) and GFP labelling the ventral median octopaminergic mVUM (median Ventral Unpaired MNs) using the *Tdc2-Gal4* driver. TJ is excluded from mVUMs. **d-f** Representative views of first instar larva VNC stained for TJ (magenta) and GFP labelling the peptidergic cells using the *Dimm-Gal4* driver. TJ is completely excluded from the larval *Dimm*$^{+}$ peptidergic population. **g, h** Representative views of first instar larva VNC stained for TJ (magenta) and GFP labelling the dopaminergic neurons using the *TH-Gal4* driver. TJ is excluded from dopaminergic neurons. **i, j** Representative views of first instar larva VNC stained for TJ (red), 5-HT (blue) and GFP labelling the monoaminergic neurons (visualized with the *vMat-GFP*, vesicular Monoamine transporter-GFP). TJ is excluded from monoaminergic neurons. In all panels, anterior of the VNC is up, midline denoted by the full line on top of the panels and the segment boundaries by the dashed lines on the right side of the panels.

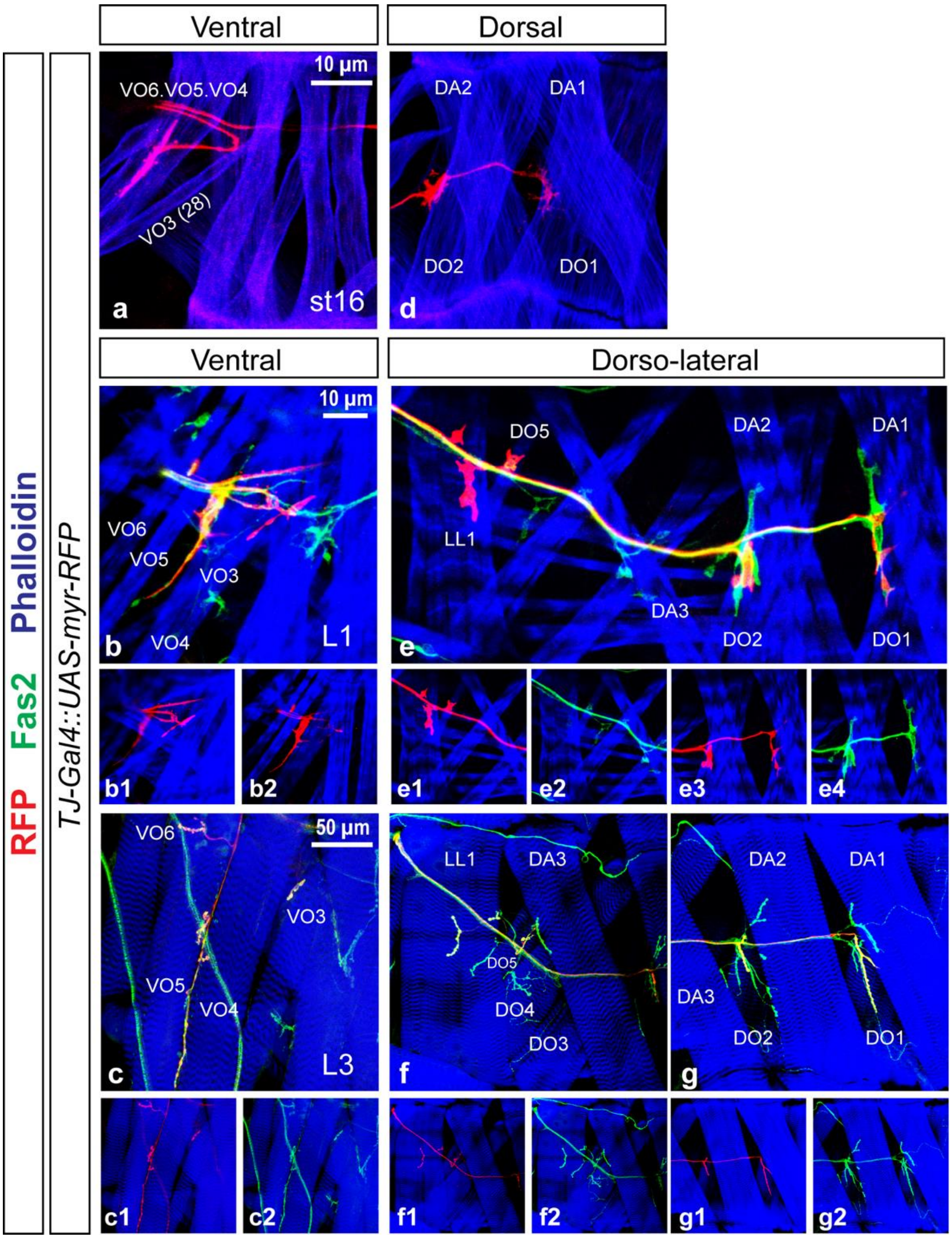

Supplementary Figure 4 TJ$^+$ MN projections remain constant from embryonic stage 16 to L3 larval stage. **a-g** Stage 16 embryo (**a**, **d**), first (**b**, **e**) and third (**c**, **f**, **g**) larval stages stained with FasII (green), *TJ-Gal4* driving a *UAS-myrRFP* (red) and Phalloidin-TX (blue). FasII labels all MN axon projections while Phalloidin-TX stains muscle fibres. TJ$^+$ MNs project through ISNd onto muscles VO3, VO4, VO5 and VO6 (**a**) and through ISNdm onto muscles DO1 and DO2 (**d**) in stage 16 embryo. TJ$^+$ MNs project through ISNd onto muscles VO3, VO4, VO5 and VO6 (**b**) and through ISNdm onto muscles DO1, DO2, DO5 and LL1 (**e**) in first instar larva. Two confocal focus plans are shown below **b** and **e** to better visualize TJ$^+$ projections on VO3, VO4, VO5 and VO6 (**b_1, b_2**), DO5 and LL1 (**e_1, e_2**) and DO1 and DO2 (**e_3, e_4**). TJ$^+$ MNs project through ISNd onto muscles VO3, VO4, VO5 and VO6 (**c**) and through ISNdm onto muscles DO5, LL1 (**f**), DO1and DO2 (**g**) in third instar larva. Two confocal focus plans are shown below **c, f** and **g** to better visualize TJ$^+$ projections on VO3, VO4, VO5 and VO6 (**c_1, c_2**), DO5 and LL1 (**f_1, f_2**) and DO1 and DO2 (**g_1, g_2**). TJ$^+$ MN projections remain unchanged through late embryonic and larval life. In all panels anterior is up and only a given region of 1 segment is shown. Anterior region of a segment is up.

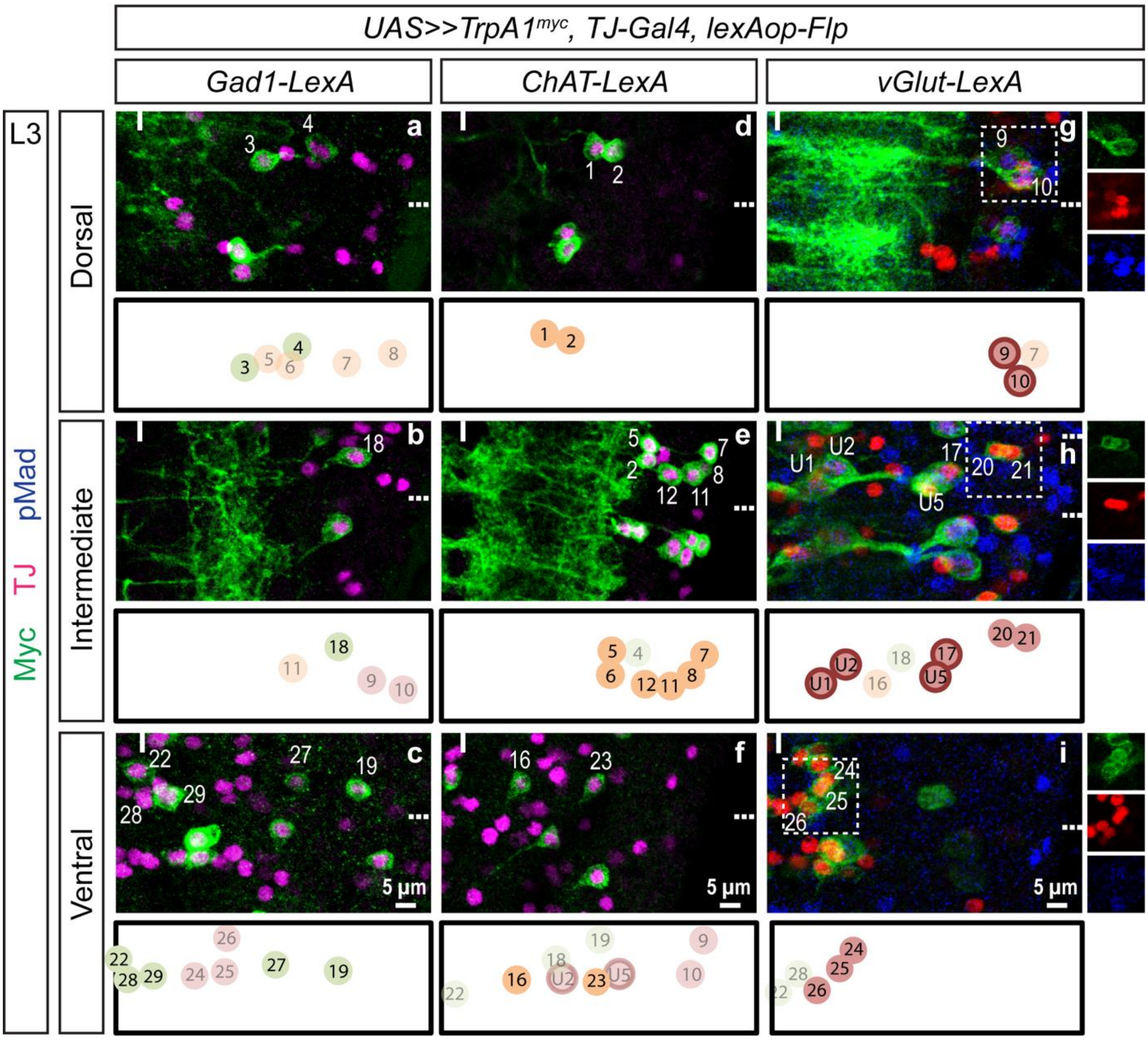

Supplementary Figure 5 TJ is expressed in highly diverse populations of interneurons in third instar larvae. **a-i** *TJ*$^{+}$ VNC cells expressing the neurotransmitters GABA, acetylcholine and glutamate are visualized using *UAS>>TrpA1-Myc, TJ-Gal4, lexAop-FLP* in combination with *Gad1-LexA* (**a-c**), *ChAT-LexA* (**d-f**) and *vGlut-LexA* (**g-i**) respectively and stained for TJ (magenta), Myc (green) and pMad (**g**, **h**, **i**) in third instar larvae. Cells are shown from dorsal to ventral positions. Midline is denoted by the full line on the left side of the panels and the segment boundaries are indicated by the dashed lines on the right side of the panels.

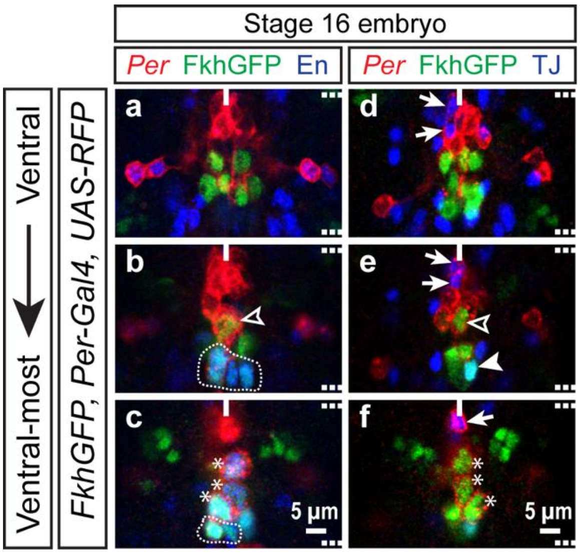

Supplementary Figure 6 In stage 16 embryo, TJ is expressed in midline MNB progeny neurons but not in H-cell sib nor iVUMs. **a-c** Staining for En (blue), *Per* (red) and FkhGFP fusion protein (green) in stage 16 embryonic VNC. Using highly stereotyped midline cell positions and *Per*, Fkh and En as markers, it is possible to precisely identify the midline cells. H-cell sib is a large *Per*$^+$/Fkh$^+$/En$^-$ cell (empty arrowhead in **b**) located in the middle of the segment and dorsally to the 3 iVUMs that are *Per*$^+$/Fkh$^+$/En$^+$ (asterisks in **c**). A group of cells located posteriorly to H-cell sib and above and posterior to the iVUMs and characterized by their expression of Fkh and En (and sometimes *Per*) are the MNB progeny neurons (circled cells in **b** and **c**). **d-f** Using stereotyped positions and *Per* and Fkh as markers, neither H-cell sib (empty arrowhead in **e**) nor the 3 iVUMs (asterisks in **f**) express TJ. Instead, at stage 16, TJ appears to be expressed in 1 MNB progeny neuron (full arrowhead in **e**). TJ is also expressed in 5 *Per*$^+$ only cells located in the anterior part of the segment (arrows in **d, e** and **f**). These *Per*$^+$ cells are not part of the *sim* domain (as seen in Fig. 9a-c), hence not part of the midline cells. Each panel represents a single segment. Segment boundaries are noted by dotted lines on the right-hand side of the panels and midline by the full line. Anterior of the VNC is up.

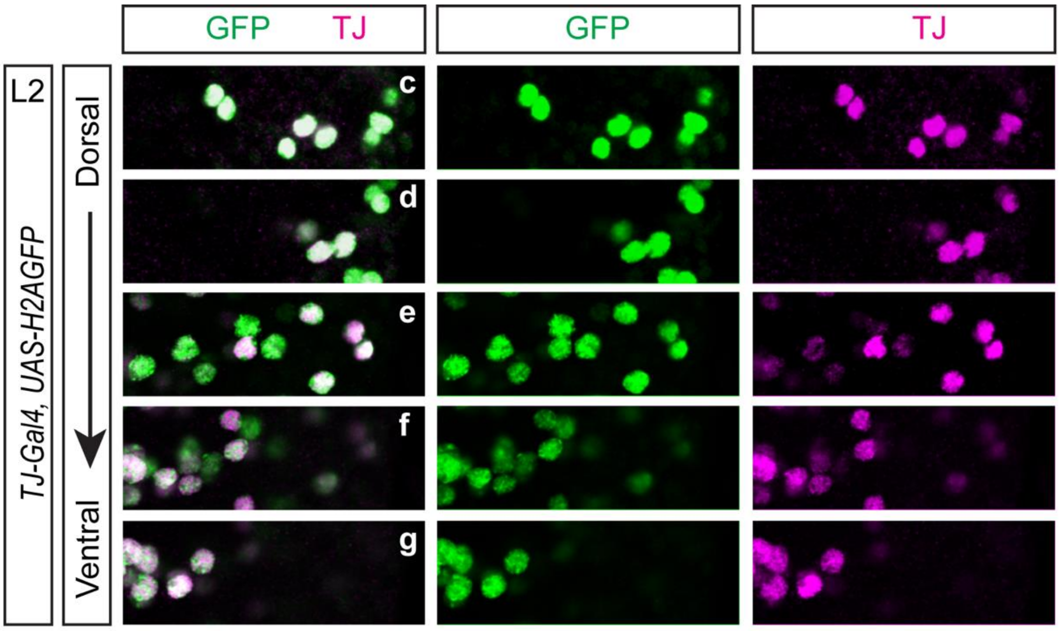

This figure contains the separated channels for the main Figure 1 panels c-g.

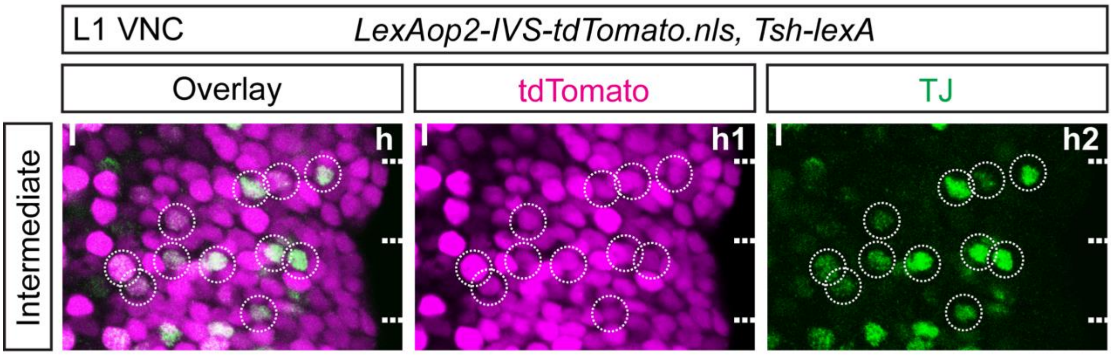

This figure contains the separated channels for the main Figure 2 panel h.

Supplementary Figure 7 TJ expression and visualization with separated channels. **Top:** This figure contains the separated channels for the main **Fig. 1** panels **c-g**. *TJ-Gal4* driving an *UAS-H2AGFP* (GFP, green) and staining for TJ (magenta) in second instar larva VNC. As easily seen with the separated channels all TJ^{+} neurons are *TJ-Gal4*$^{+}$. Numbering of TJ^{+} cells has been omitted for clarity. **Bottom:** This figure contains the separated channels for the main **Fig. 2** panel **h**. *Tsh-LexA* driving an *nls-tdTomato* (**h1**, magenta) and staining for TJ (**h2**, green) in first instar larva VNC. Here also all TJ^{+} neurons (green) are clearly *Tsh-lexA*$^{+}$ (magenta). In both cases note the variable levels of expression of TJ in TJ^{+} neurons.
