# Supplementary material for: A GABAergic Maf-expressing interneuron subset regulates the speed of locomotion in Drosophila
Source: Nat Commun. 2019 Oct 22;10:4796. doi: 10.1038/s41467-019-12693-6 (PMC6805931; doi:10.1038/s41467-019-12693-6)
Supplement: Supplementary file 3 — Reporting summary [file 41467_2019_12693_MOESM3_ESM.pdf]

natureresearch

Corresponding author(s): Alain Garcès

Last updated by author(s): 2019.09.12

Reporting Summary

Nature Research wishes to improve the reproducibility of the work that we publish. This form provides structure for consistency and transparency in reporting. For further information on Nature Research policies, see Authors & Referees and the Editorial Policy Checklist.

Statistics

For all statistical analyses, confirm that the following items are present in the figure legend, table legend, main text, or Methods section.

n/a	Confirmed	
☐	☒	The exact sample size (*n*) for each experimental group/condition, given as a discrete number and unit of measurement
☐	☒	A statement on whether measurements were taken from distinct samples or whether the same sample was measured repeatedly
☐	☒	The statistical test(s) used AND whether they are one- or two-sided<br>*Only common tests should be described solely by name; describe more complex techniques in the Methods section.*
☐	☒	A description of all covariates tested
☐	☒	A description of any assumptions or corrections, such as tests of normality and adjustment for multiple comparisons
☐	☒	A full description of the statistical parameters including central tendency (e.g. means) or other basic estimates (e.g. regression coefficient) AND variation (e.g. standard deviation) or associated estimates of uncertainty (e.g. confidence intervals)
☐	☒	For null hypothesis testing, the test statistic (e.g. F, t, r) with confidence intervals, effect sizes, degrees of freedom and P value noted<br>*Give P values as exact values whenever suitable.*
☒	☐	For Bayesian analysis, information on the choice of priors and Markov chain Monte Carlo settings
☒	☐	For hierarchical and complex designs, identification of the appropriate level for tests and full reporting of outcomes
☒	☐	Estimates of effect sizes (e.g. Cohen's *d*, Pearson's *r*), indicating how they were calculated

Our web collection on statistics for biologists contains articles on many of the points above.

Software and code

Policy information about availability of computer code

Data collection	-Confocal images were acquired on a Zeiss LSM700 confocal microscope with 40x or 63x objectives.<br>-For locomotor behavior the crawling larvae were tracked in real-time using the MWT (Multi-Worm Tracker) software (N. A. Swierczek, A. C. Giles, C. H. Rankin, R. A. Kerr, High-throughput behavioral analysis in C. elegans. Nature Methods 8, 592 (2011)).
Data analysis	Confocal images were treated using Zen (Zeiss) and ImageJ softwares, cropped in Photoshop (Adobe) and assembled in Illustrator (Adobe).

For manuscripts utilizing custom algorithms or software that are central to the research but not yet described in published literature, software must be made available to editors/reviewers. We strongly encourage code deposition in a community repository (e.g. GitHub). See the Nature Research guidelines for submitting code & software for further information.

Data

Policy information about availability of data

All manuscripts must include a data availability statement. This statement should provide the following information, where applicable:

- Accession codes, unique identifiers, or web links for publicly available datasets
- A list of figures that have associated raw data
- A description of any restrictions on data availability

The datasets generated during and/or analysed during the current study are available from the corresponding author on reasonable request.

Field-specific reporting

Please select the one below that is the best fit for your research. If you are not sure, read the appropriate sections before making your selection.

☒ Life sciences ☐ Behavioural & social sciences ☐ Ecological, evolutionary & environmental sciences

For a reference copy of the document with all sections, see nature.com/documents/nr-reporting-summary-flat.pdf

Life sciences study design

All studies must disclose on these points even when the disclosure is negative.

Sample size	No sample-size calculations were performed. Sample size was determined based on previous work already published in our field. In our study the number of larvae analysed in each genetic condition is equal or superior to the number of larvae that were analysed in similar and already published studies. Sample size was determined to be adequate based on the magnitude and consistency of quantifiable differences between the different genetic conditions tested.
Data exclusions	No data were excluded from the analyses.
Replication	All attempts at replication were successful.
Randomization	For behaviour essays we randomly picked up larvae of a given genotype (the number of larvae is indicated within each figures) from a large population of larvae carrying the same genotype (n>100). The sex of the larvae under investigation was not determined thus not taken into consideration.
Blinding	The investigators were not blinded to group allocation. Larvae behaviors were tracked in real-time using the MWT (Multi-Worm Tracker) software (N. A. Swierczek, A. C. Giles, C. H. Rankin, R. A. Kerr, High-throughput behavioral analysis in C. elegans. Nature Methods 8, 592 (2011)). From the MWT tracking data the key parameters of larval motion were computed using specific choreography variables (part of the MWT software package). The average speed of larvae in mm per second, the average body curve (in radians as a proxy for larval head-casting), and average crab speed (sideways rolling speed in mm per second) performed by the larvae during 1 minute was determined.

Reporting for specific materials, systems and methods

We require information from authors about some types of materials, experimental systems and methods used in many studies. Here, indicate whether each material, system or method listed is relevant to your study. If you are not sure if a list item applies to your research, read the appropriate section before selecting a response.

Materials & experimental systems

n/a	Involved in the study	
☐	☒	Antibodies
☒	☐	Eukaryotic cell lines
☒	☐	Palaeontology
☐	☒	Animals and other organisms
☒	☐	Human research participants
☒	☐	Clinical data

Methods

n/a	Involved in the study	
☒	☐	ChIP-seq
☒	☐	Flow cytometry
☒	☐	MRI-based neuroimaging

Antibodies

Antibodies used	The list of antibodies used is provided in Table 1 in the manuscript.
Validation	For each antibody the validation data is provided in the publication that we have referred to and by the companies that commercialize the antibodies (see Table 1).

Animals and other organisms

Policy information about studies involving animals; ARRIVE guidelines recommended for reporting animal research

Laboratory animals	Drosophila melanogaster. The names, genotypes and sources of the different Drosophila stocks used in the study are provided in Table 2.
Wild animals	The study did not involve wild animals.
Field-collected samples	The study did not involve samples collected from the field.

Ethics oversight | No applicable for studies on Drosophila melanogaster.

Note that full information on the approval of the study protocol must also be provided in the manuscript.
