# Supplementary material for: A GABAergic Maf-expressing interneuron subset regulates the speed of locomotion in Drosophila
Source: Nat Commun. 2019 Oct 22;10:4796. doi: 10.1038/s41467-019-12693-6 (PMC6805931; doi:10.1038/s41467-019-12693-6)
Supplement: Supplementary file 4 — Description of Additional Supplementary Files [file 41467_2019_12693_MOESM4_ESM.pdf]

Description of Additional Supplementary Files

File Name: Supplementary Movie 1
Description: Visualization of TJ-expressing (TJ^{+}) neurons in L1 Ventral Nerve Cord. 3D in depth color projection showing the respective dorsal to ventral locations of TJ+ neurons. Color code reads as follow: Yellow indicate dorsal most positions while dark blue ventral most locations.

File Name: Supplementary Movie 2
Description: Silencing of the entire TJ+ neuronal population in a L1 larva carrying TJ-Gal4, UAS-shi^{ts} .

File Name: Supplementary Movie 3
Description: Activation of the entire TJ^{+} neuronal population in a L1 larva carrying TJGal4, UAS-TrpA1.

File Name: Supplementary Movie 4
Description: Activation of the TJ^{+} neurons in the VNC only in a L1 larva carrying TJ-Flp, LexAop>Stop>dTrpA1, Tsh-LexA.

File Name: Supplementary Movie 5
Description: Control L1 larva carrying LexAop>Stop>dTrpA1, CQ2-LexA.

File Name: Supplementary Movie 6
Description: Activation of TJ^{+} motoneurons in a L1 larva carrying TJ-Flp, LexAop>Stop>dTrpA1, CQ2-LexA.

File Name: Supplementary Movie 7
Description: Recording of control L3 larvae using MWT (Multi-Worm Tracker) carrying TJFlp, LexAop>Stop>dTrpA1 at 23°C.

File Name: Supplementary Movie 8
Description: Recording of control L3 larvae using MWT (Multi-Worm Tracker) carrying TJFlp, LexAop>Stop>dTrpA1 at 31°C.

File Name: Supplementary Movie 9
Description: Activation of TJ^{+} /Cholinergic+ interneurons in a L3 larva carrying TJ-Flp, LexAop>Stop>dTrpA1, ChAT-LexA.

File Name: Supplementary Movie 10
Description: Recording of L3 larvae using MWT (Multi-Worm Tracker) upon activation of TJ^{+} /Cholinergic^{+} interneurons using TJ-Flp, LexAop>Stop>dTrpA1, ChAT-LexA.

File Name: Supplementary Movie 11
Description: Close up of a L3 larva using MWT (Multi-Worm Tracker) upon activation of TJ^{+} /Cholinergic^{+} interneurons using TJ-Flp, LexAop>Stop>dTrpA1, ChAT-LexA.

File Name: Supplementary Movie 12
Description: Activation of TJ^{+} /Glutamatergic^{+} neurons in a L3 larva carrying TJ-Flp, LexAop>Stop>dTrpA1, vGlut-LexA.

File Name: Supplementary Movie 13
Description: Close up of 2 L3 larvae using MWT (Multi-Worm Tracker) upon activation of TJ^{+} /Glutamatergic^{+} using TJ-Flp, LexAop>Stop>dTrpA1, vGlut-LexA.

File Name: Supplementary Movie 14
Description: Control L3 larva carrying TJ-Flp, LexAop>Stop>dTrpA1 at 23°C and 31°C.

File Name: Supplementary Movie 15
Description: Activation of TJ^{+} /GABAergic^{+} neurons in a L3 larva carrying TJ-Flp, LexAop>Stop>dTrpA1, Gad1-LexA.

File Name: Supplementary Movie 16
Description: Recording of L3 larvae using MWT (Multi-Worm Tracker) upon activation of TJ^{+} /GABAergic^{+} interneurons using TJ-Flp, LexAop>Stop>dTrpA1, Gad1-LexA.

File Name: Supplementary Movie 17
Description: Activation of TJ^{+} /Period^{+} interneurons in a L3 larva carrying TJ-Flp, LexAop>Stop>dTrpA1, Per-LexA.

File Name: Supplementary Movie 18
Description: Recording of L3 larvae using MWT (Multi-Worm Tracker) upon activation of TJ^{+} /Period^{+} interneurons using TJ-Flp, LexAop>Stop>dTrpA1, Per-LexA.

File Name: Supplementary Movie 19
Description: Control L3 larva carrying TJ-Flp, LexAop>Stop>dTrpA1, Per-Gal4, UASLexA^{DBD} at 23°C and 31°C.

File Name: Supplementary Movie 20
Description: Activation of TJ^{+} /Period^{+} /Gad1^{+} interneurons in a L3 larva carrying TJ-Flp, LexAop>Stop>dTrpA1, Per-Gal4, UAS-LexA^{DBD}, Gad1^{AD} .

File Name: Supplementary Movie 21
Description: Recording of L3 larvae using MWT (Multi-Worm Tracker) upon activation of TJ^{+} /Period^{+} /Gad1^{+} interneurons using TJ-Flp, LexAop>Stop>dTrpA1, Per-Gal4, UAS-LexA^{DBD}, Gad1^{AD} .

File Name: Supplementary Movie 22
Description: 3D reconstruction of a first instar larva VNC showing the morphology of Ladder-d neurons visualized using the split Gal4 line JRC-SS00863 driving 20XUAS-6XGFP and stained for FasII and GFP.
